# Slime molds as a valuable source of antimicrobial agents

**DOI:** 10.1186/s13568-021-01251-3

**Published:** 2021-06-23

**Authors:** Vida Tafakori

**Affiliations:** Department of Cell and Molecular Biology, Faculty of Science, Kharazmi University, Tehran, Iran

**Keywords:** Multidrug, Resistance, Antibiotics, Natural products, *Myxomycetes*

## Abstract

Given the emerging multidrug-resistant pathogens, the number of effective antimicrobial agents to deal with the threat of bacterial and fungal resistance has fallen dramatically. Therefore, the critical solution to deal with the missing effective antibiotics is to research new sources or new synthetic antibiotics. Natural products have different advantages to be considered antimicrobial agents. There are different natural sources for antimicrobial agents, such as bacteria, fungi, algae, slime molds, and plants. This article has focused on antibiotics from slime molds, especially *Myxomycetes*. The reason why slime molds have been chosen to be studied is their unique bioactive metabolites, especially over the past couple of decades. Some of those metabolites have been demonstrated to possess antibiotic activities. Hence, this article has focused on the potential of these creatures as an alternative source of antibiotics.

## Introduction

In 1940, Edward Abraham and Ernest Chain at Oxford University in the United Kingdom affected the *Escherichia coli* extraction on the penicillin and proved the existence of a penicillinase, which was able to inactivate penicillin, warning about intrinsic antibiotic resistance. This happened soon after the discovery of penicillin in 1928 when the drug was not being used yet (Amenu 2014). Antibiotics, these “wonder drugs,” were used to treat injured soldiers and wounded civilians during World War II. However, after penicillin came into clinical use, the additional bacteria developed the ability to resist other antibiotics, unfortunately. Nowadays, the appearance of multidrug-resistant bacteria (Table 1) has made antimicrobial resistance a major threat to global health and development. The emerging multidrug-resistant pathogens have dramatically lowered the number of effective antimicrobial agents to deal with the threat of bacterial and fungal resistance. The provisional leading cause-of-death rankings for 2020 indicate COVID-19 as the third leading cause of death in the US behind heart disease and cancer (Ahmad et al. 2021). Thus, infectious diseases are still concerned among the most important global social challenge of the world.

**Table 1:** Multidrug resistant bacteria observed in the community (Duin and Paterson 2016)

Multidrug resistant (MDR) phenotype	Epidemiologic setting of community-onset infections	Major antibiotics resistance mechanisms
Methicillin-resistant *S. aureus* (MRSA)	Household colonization; farm animal exposure (emerging)	mecA, mecC
Vancomycin-resistant *Enterococci* (VRE)	Typically health care-associated	vanA, vanB
Carbapenem-resistant *Acinetobacter baumannii* (CRAB)	Extremely rare	Carbapenemases
Multi-drug resistant *Pseudomonas aeruginosa*	Extremely rare	Extended-spectrum beta-lactamases, carbapenemases, and efflux systems
Extended-spectrum beta-lactamase (ESBL)-producing *Enterobacteriaceae*	Endemic in Asia; in low-prevalence areas travel to Asia	Extended-spectrum beta-lactamases, and efflux systems
Carbapenem-resistant *Enterobacteriaceae* (CRE)	Rare at present; emerging in India and China	Carbapenemases

Reports estimate that, by 2050, 10 million people will die every year with an estimated economic cost of $100 trillion due to antimicrobial resistance unless a global response is mounted to this problem (Simpkin et al. 2017). Accordingly, the critical solution to deal with the missing effective antibiotics is to study on new sources or new synthetic antibiotics.

In the antimicrobial resistance process, microbial cells have developed a complex and redundant barrier to penetration, enzymes to destroy or modify, and pumps to efflux the antibiotic compounds. Hence, between natural products and synthetic ones, the former is preferable since as the microbial cells evolve and gain the ability to develop resistance against antimicrobial agents, natural products also evolve in nature. Another reason is that in the drug discovery sector, synthetic antibiotics are most favor human bioactivity, not suitable for microbial biology (Wright 2014). On the other hand, natural products reflect the true chemical diversity (extensive functional group chemistry and chirality), which does not exist in synthetic products (Phillipson 2007). On the other hand, for some natural products, the apparent lack of industrial interest may be due to the inherent time-consuming working process with natural products, difficulties in access and supply, costs of collection, extraction, and isolation, and standardization limitations of crude drugs (Phillipson 2007; Harvey 2008). However, despite all these problems, their advantages still make natural products the preferable choice for antimicrobial agent sources.

## Natural sources of antimicrobial agents

Although antibiotics generally refer to antibacterials, since the term has not exactly been defined, it encompasses a wide variety of agents, including antibacterial, antifungal, antiviral, and antiparasitic drugs (Cheesbrough 2006).

There are different natural sources of antimicrobial agents, such as filamentous bacteria actinomycetes (more than 50% of all antibiotics are produced by these microorganisms), non-filamentous bacteria (10–15%) (Demain 2014; Salehghamari et al. 2015), microscopic fungi (about 20% of all antibiotics are produced by filamentous fungi) (Demain 2014), slime molds, algae (Ibrahim and Lim 2015; Pane et al. 2015), plants (Abreu et al. 2012; Tafakori and Nasiri 2018; Eftekhar et al. 2005), animals (Primon-Barros and José Macedo 2017; Han et al. 2011), and other natural sources.

There are different approaches for effective drug discovery programs: bioactive-guided screening, chemical screening, target-oriented screening, the use of unexplored strains, genome mining (using next-generation DNA sequencing), and activation of silent gene clusters (Wohlleben et al. 2016). Among different natural sources mentioned above, some have received extra attention, such as actinomycetes, microscopic fungi, and plants. However, the others, such as slime molds, have not been taken into account and can be considered unexplored strains. Therefore, this article has focused on the potential of these creatures as an alternative source of antibiotics.

## Slime molds

Slime molds are protists with two stages in their life cycles. In one stage, they behave like protozoa (amoeba) while acting like fungi in the other. In the protozoan phase, they engulf food particles, other microbes and consuming decaying vegetation. These microorganisms are included in two groups: acellular slime molds (*Myxogastria*) and cellular slime molds (*Dictyostelium*) (Willey et al. 2014). Recently, slime molds have been known as good resources of natural compounds with bioactivity. Dembitsky et al. (2005) reported almost 100 natural compounds from only one of them, namely *Myxomycetes*. Some of these metabolites were bioactive compounds. The physiology and biochemistry of *Myxomycetes* have considered, but their secondary metabolites have remained virtually unknown. Some of these metabolites have been shown to possess antioxidant, cytotoxic, and antibacterial activities. This review focused on bioactive compounds with antibiotic activity.

## Slime molds types

### Acellular slime molds

The life cycle of this group of slime molds encompasses two strikingly different trophic (feeding) stages. The plasmodium structure, consisting of a distinctive multinucleate mass, involves as many as 10,000 dividing nuclei without individual cell membranes. This colorful protoplasm creeps along in amoeboid fashion over moist, cool, and shaded places, such as within cracks of decomposing wood, underneath the partially decayed bark of tree trunks and stumps, or other organic matter in high humidity condition, and in leaf debris on the forest floor which degrades and feeding by endocytosis (Willey et al. 2014; Dembitsky et al. 2005). The other stage of its life cycle starts when plasmodium mass is starved or dried. At this time, the plasmodium typically differentiates and develops fruiting bodies containing stalks with cellulose walls. Fruiting bodies are resistant to environmental stresses and contain spores (Willey et al. 2014).

**Fig. 1:**
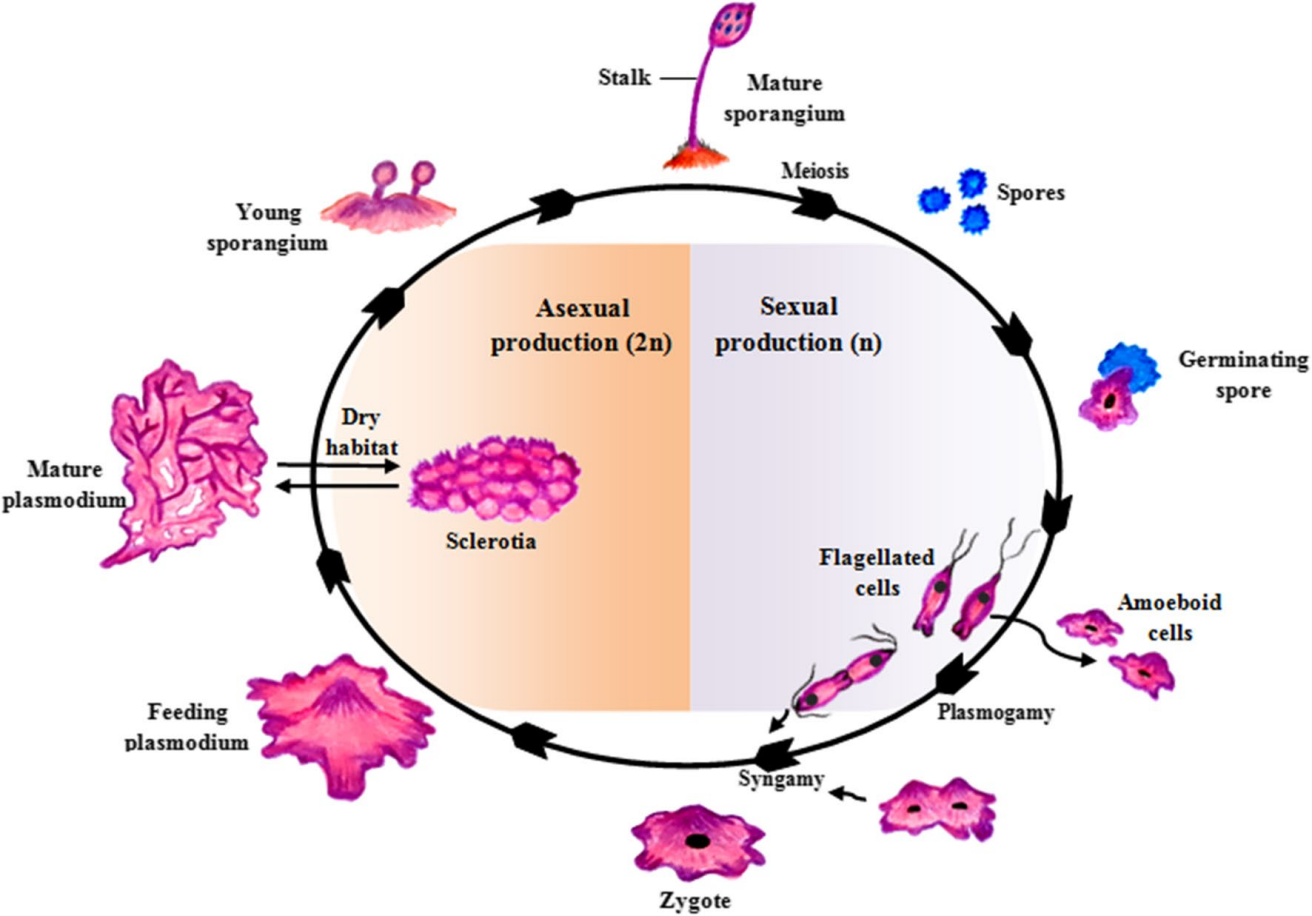
The life cycle of a plasmodial slime mold includes sexual and asexual reproduction

The spores are, for most species, apparently wind-dispersed (Dembitsky et al. 2005). Under favorable conditions, spores germinate, and haploid amoebae flagellate cells are released. These cells fuse, forming zygotes. Then, these diploid cells feed, and nuclei divide asynchronously to form the multinucleate plasmodium (Willey et al. 2014). Figure 1 illustrates the life cycle of acellular slime molds.

**Fig. 2:**
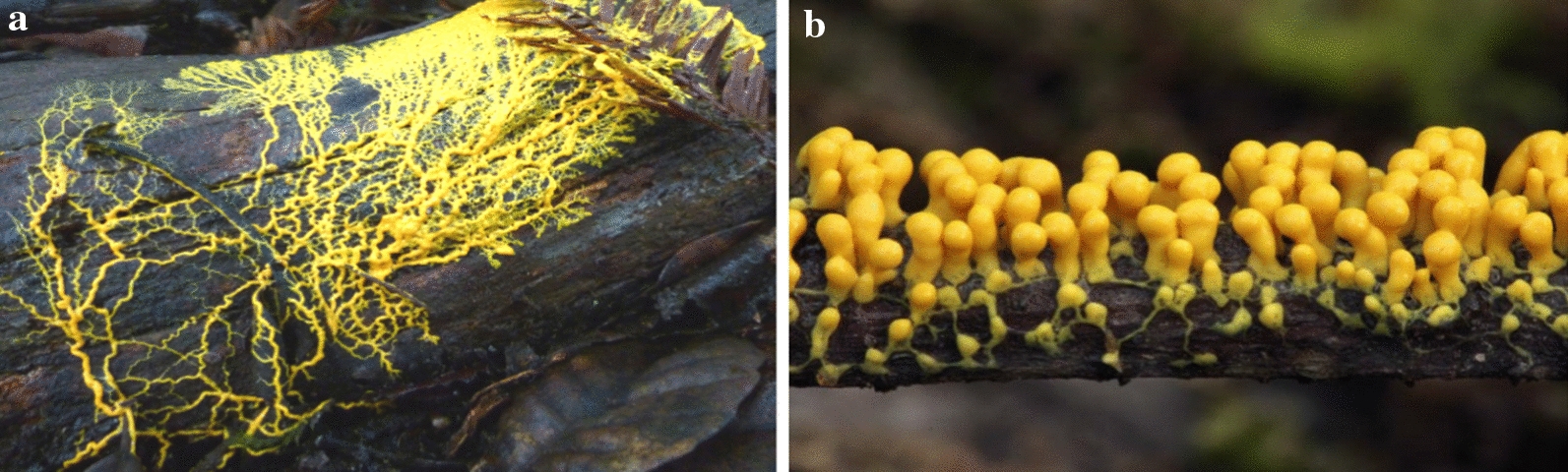
**a** Plasmodium of the slime mold *Physarum* sp. **b** Sporangia of *Physarum polycephalum* (https://calphotos.berkeley.edu)

*Myxomycetes* are among the well-known classes of acellular slime molds that produce a large number of metabolites (Dembitsky et al. 2005). These metabolites were directly extracted from fruiting bodies. Thus, in vitro cultivation of *Myxomycetes* is a solution to produce a massive amount of fruiting bodies. Among *Myxomycetes*, most in vitro cultured species belong to the genera *Physarum* and *Didymium* (Dembitsky et al. 2005)*.* Figure 2 illustrates the plasmodium form and sporangia of the *Physarum*.

### Cellular slime molds

**Fig. 3:**
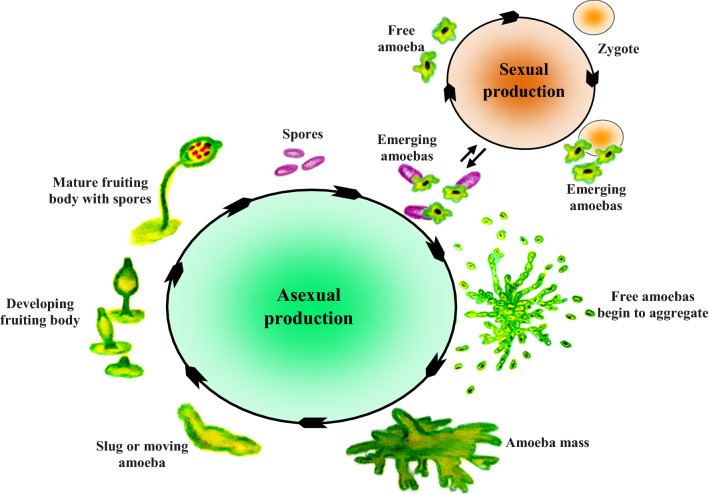
Life cycle of a cellular slime mold

This group of slime molds has a complex life cycle involving true multicellularity, called pseudoplasmodium, which differs from the acellular slime mold’s true plasmodium. It consists of an aggregated mass of individual amoebas that are able to migrate as large, motile, multicellular slugs. Actually, when the starved cells release molecular signals, cAMP, and a specific glycoprotein, the other cells sense these molecules and aggregate around the signal-producing cells (Willey et al. 2014). Ultimately, some of the amoebae cells sacrifice themselves and differentiate to the dead vacuolated stack that produces fruiting bodies containing countless and unicellular spores. Under favorable conditions, released spores germinate and release separate vegetative amoebae that feed on bacteria and yeasts. After running out of food and starvation, the amoebae aggregate to form pseudoplasmodium again (Bonner and Lamont 2005). Figure 3 shows the life cycle of cellular slime mold, *Dictyostelium discoideum*, an attractive model species.

### Antibiotics from slime molds

**Fig. 4:**
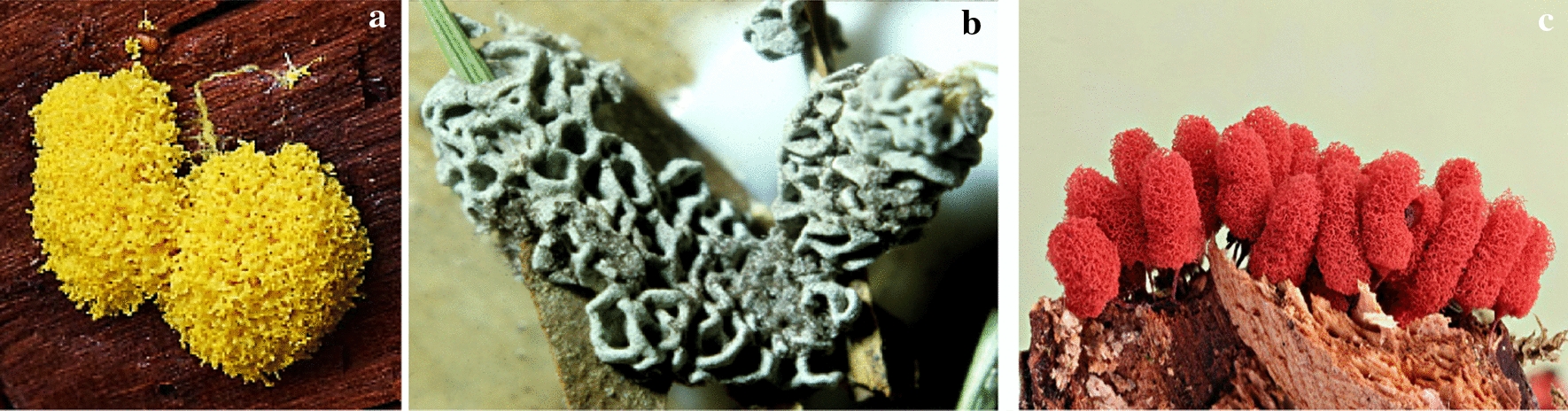
**a*** Fuligo septica*, **b*** Physarum gyrosum ***c*** Arcyria denudata* (https://www.naturepl.com, https://www.discoverlife.org)

This part of the article focuses on antimicrobial agents from slime molds according to the dates of reports. The first report on antibiotics from slime molds, has been published more than 73 years ago. In 1948, two anthraquinone acids, derivatives of an aromatic organic compound as pigment, were isolated from *Fuligo septica* (Fig. 4a) with antibiotic activity (Loquin and Prevot 1948). These compounds are aromatic yellow pigment of *F. septica.* According to Sobels (1950), mucous secretions or aqueous extracts of *Licea flexuosa* plasmodium in pure culture and in association with *Torulopsis laurentii* (a yeast species) inhibited the growth of *Cladosporium herbarum*, *Penicillium* sp., certain bacteria, and yeasts.

Antibacterial products from *Physarum gyrosum* plasmodium (Fig. 4b) on agar plate have been extracted and partially purified. It had effected on *S. aureus, B. cereus, B. subtilis*, *E. coli,* and *P. aeruginosa* (Considine and Mallette 1965). A butanolic and fractionated (pure heterocyclic antibiotic D-1) extract of plasmodium form of *Physarum gyrosum* had antibacterial effects against gram-positive bacteria, *S. cerevisiae*, Mycobacterium 156, *Torulopsis sphaerica* and intermediate effect on *E. coli.* Pure antibiotic was effective against *B. cereus* (Schroeder and Mallette 1973).

Steglich et al. (1980) extracted and isolated pigment from the fruiting bodies of the slime mold, *Arcyria denudata* (Fig. 4c) with methanol, which makes them red and yellow. These main pigments were bisindolylmaleimides and named arcyriarubin B (1), C (2), arcyriaflavin B (3), C (4), and arcyrioxepin A (5), among which compounds 1, 2, and 5 showed medium inhibiting zone against *B. brevis* and *B. subtilis* in the plate diffusion assay.

**Fig. 5:**
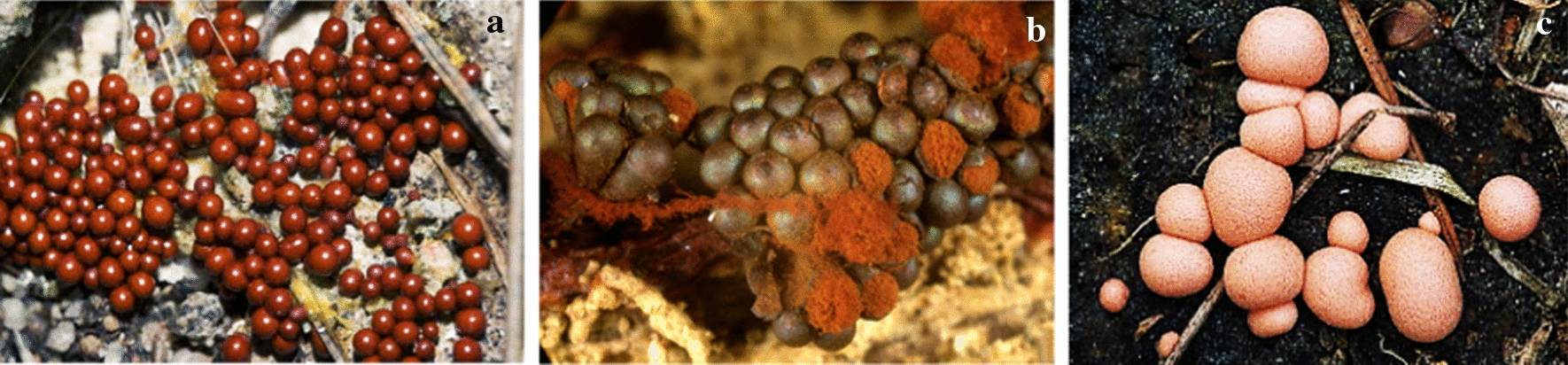
**a*** Leocarpus fragilis, ***b*** Metatrichia vesparium, ***c*** Lycogala epidendrum *(https://www.discoverlife.org, https://www.naturepl.com)

Casser et al. (1987) also isolated a yellow pigment, Fuligorubin A, from the plasmodia of *Fuligo septica,* which is the first tetramic acid derivative to be isolated from slime molds. These components have the butenolide structure with antibiotics, antitumor, and mycotoxins properties. The acyltetramic acids are also responsible for the orange-yellow color of plasmodia from *Leocarpus fragilis* (Fig. 5a). These components have previously been isolated from streptomycetes and fungi. Three possible roles are attributed to acyltetramic acids: first, as the protector of plasmodia of slime molds against the attack of microorganisms; second, as photoreceptors; and third, as metal chelating agents (Steglich 1989).

Kopanski et al. (1987) isolated a red pigment of the fruit bodies, namely vesparione, a naphtha[2,3-b]pyrandione derivative, from another myxomycete, *Metatrichia vesparium* (Fig. 5b). This pigment had antibiotic properties. Lycogarubins A-C, three novel dimethyl pyrrole dicarboxylate, were isolated from *Myxomycetes* *Lycogala epidendrum* (Fig. 5c). Lycogarubin C showed moderate anti-HSV-I virus activity (Hashimoto et al. 1994). Wang et al. (2017) also reported that lycogalinosides A and B, two compounds isolated from *L. epidendrum,* showed inhibitory activities against gram-positive bacteria. Lycogarubins A-C are pigment and polycyclic compounds.

Chiappeta et al. (1999) examined the influence of methanol pH on the extraction of the fruiting bodies of *F. septica*. The extracts were obtained at pH 2 and 7, but not at pH 9, had antimicrobial activity against *S. aureus*, *B. subtilis, M. luteus* and *C. albicans*. AB0022A, a highly substituted aromatic and a Dibenzofuran, was isolated from the *Dictyostelium purpureum* K1001, a cellular slime mold, and exhibited the growth inhibition of different gram-positive bacteria, such as *S. aureus* and *S. pyogenes* (Sawada et al. 2000).

**Fig. 6:**
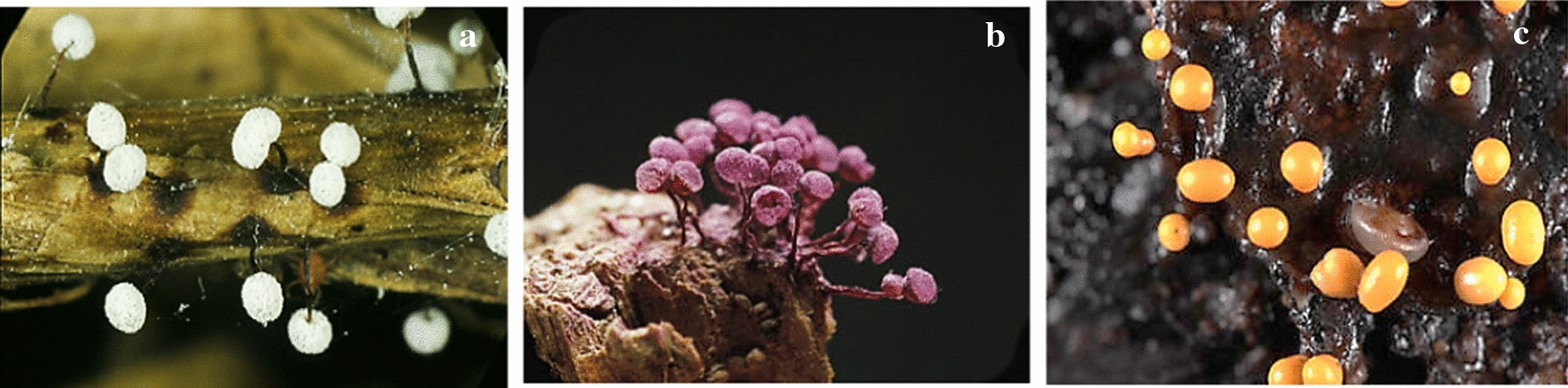
**a** *Didymium bahiense*, **b** *Cribraria purpurea*, **c*** Trichia varia* (https://www.discoverlife.org, https://www.naturepl.com)

Misono et al. (2003) isolated a new glycerolipid, namely Bahiensol, from the plasmodium form of myxomycete *Didymium bahiense* var*. bahiense* (Fig. 6a). The molecular formula of Bahiensol was C_19_H_40_O_5_, and it showed non-significant activity against *B. subtilis.* The inhibition zone diameter was 12.5 mm for 500 mg Bahiensol per paper disc (8 mm in diameter).

Naoe et al. (2003) isolated Cribrarione A from a myxomycete *Cribraria purpurea* (Fig. 6b). This compound was a dihydrofuran naphthoquinone pigment, and its structure was determined by mass spectrometric data. In its Heteronuclear Multiple Bond Correlation spectrum, the 1H–13C long-range couplings constants an intramolecular hydrogen bond were revealed. Cribrarione A showed antibacterial activity against *B. subtilis.* The diameter of the inhibition zones was 11 mm (including 8 mm paper disc) at 5 µg per paper disc.

**Fig. 7:**
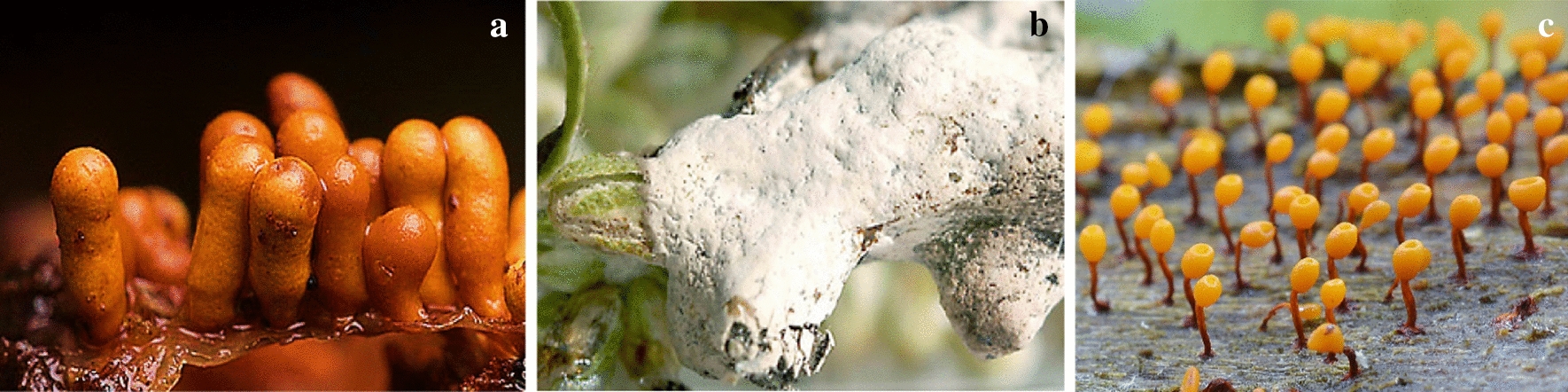
**a** *Trichia favoginea*, **b** *Fuligo cinerea*, **c*** Physarella oblonga* (http://www.sanamyan.com/myxomycetes, https://www.discoverlife.org, http://mycoweb.ru)

**Fig. 8:**
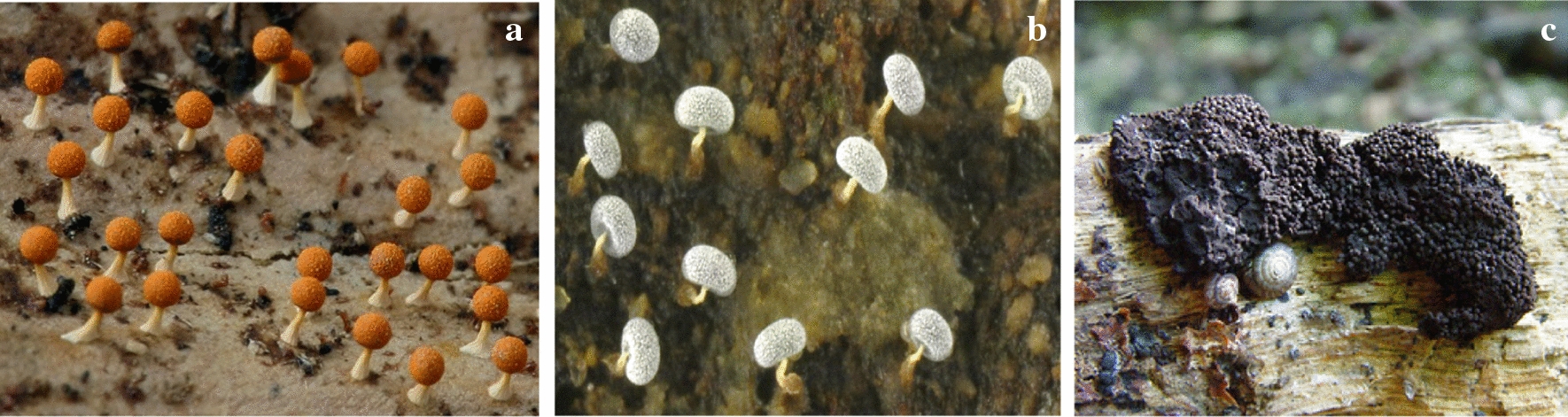
**a** *Physarum melleum.* **b*** Physarum album,* **c*** Symphytocarpus amaurochaetoides.* (https://www.discoverlife.org)

Rare fatty acids with Δ^5,9^-position of two double bonds have been identified in two *Myxomycetes*, *Trichia varia* (Fig. 6c) and *T. favoginea* (Fig. 7a) for the first time (Dembitsky et al. 2005). The compounds with similar structures were particularly active against Gram-positive bacteria, such as *S. aureus* and *S. faecalis*, but this was not true about gram-negative bacteria (Carballeira et al. 1997). Kehokorins A, a polycyclic pigment, extracted from *Trichia favoginea* var. *persimilis*, exhibited inhibition zone with 10.1 mm at 50 µg per disc (8 mm in diameter) against *S. aureus* but had no activity against *B. subtilis* at that concentration (Kaniwa et al. 2006). *Fuligo cinerea* (Fig. 7b) produces an unusual metabolite with glycosidic dibenzofuran structure, Fulicineroside. It has exhibited a high level of activity against Gram-positive bacteria (Rezanka et al. 2005). The plasmodial extracts of *Physarella oblonga* (Fig. 7c) showed an antiparasitic activity against epymastigote forms of *Trypanosoma cruzi,* while one of the used controls had significantly lower activity. On the other hand, myxomycete *Physarum melleum,* (Fig. 8a) showed antifungal activity against the phytopathogen *F. oxysporum* (Herrera et al. 2011)***.***

A crude extract of *F. septica,* was shown to have significant inhibitory effects on *E. coli* and *B. subtilis*, with inhibition rates of 68.00% and 59.45%, respectively*.* However, this extract had only a minor effect on *S. aureus* and *S. typhimurium* and no effect on *P. pyocyaneum* (Jiang et al. 2014). A secondary metabolite of SteelyA polyketide synthase, 4-Methyl-5-pentylbenzene-1,3-diol (MPBD), controlled the aggregation of individual cells and spore maturation of *Dictyostelium discoideum.* This compound had antimicrobial activities toward *E. coli* and *B. subtilis* (Murata et al. 2016)*.* Exopolysaccharides (EPS) from *Ph. oblonga* and *Ph. polycephalum* showed significant antimicrobial activities, especially against *Candida albicans* with an inhibition zone of 20 mm. MICs of this yeast were found to be 2560 µg/ml for EPS from *Phy. Oblonga,* and 1280 µg/ml for EPS from *Ph. Polycephalum* (Huynh et al. 2017).

In 2018, Sevindik et al. reported antibacterial and antifungal activity of ethanolic, methanolic and dichloromethanic extracts of sporophore stage *Physarum album* (Fig. 8b) with the highest activity against *Acinetobacter baumannii*. They used modified agar dilution method on *Staphylococcus aureus*, *S*. *aureus* MRSA, *Enterococcus faecalis*, *Escherichia coli*, *Pseudomonas aeruginosa*, *Klebsiella pneumoniae*, *Acinetobacter baumannii*, *Candida albicans*, *C*. *krusei*, and *C*. *glabrata*.

**Fig. 9:**
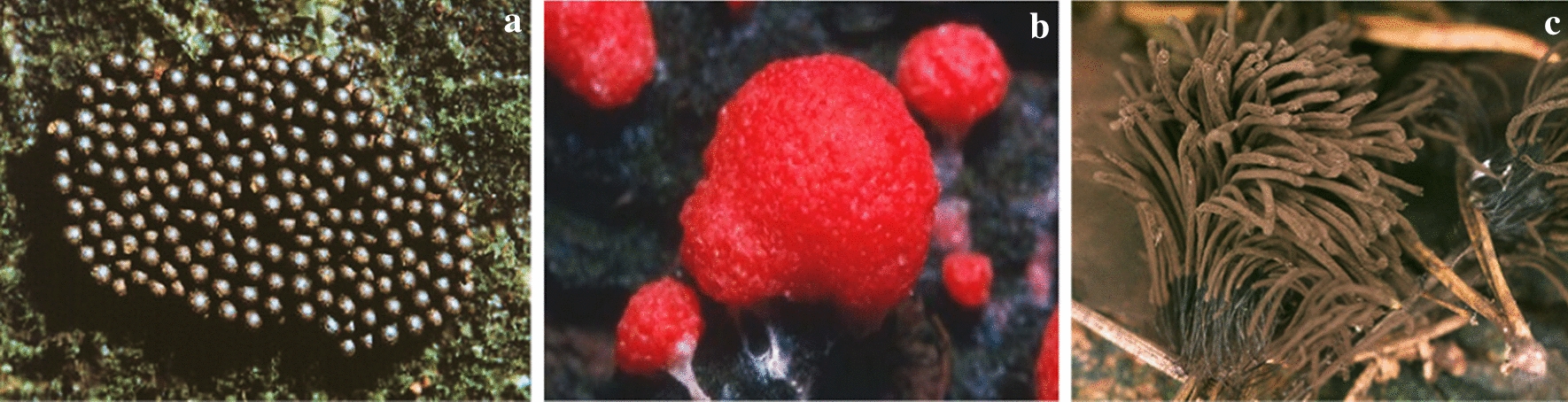
**a*** Lindbladia tubulina* Fr, **b** *Tubifera ferruginosa* (Batsch) Gmelin, **c** *Stemonitis fusca* Roth (https://www.discoverlife.org)

The chlorinated alkylphenone differentiation-inducing factors (DIFs) induce stalk cell developing in *D. discoideum*. These compounds and their derivatives exhibited several biological activities, including antibacterial properties. Many of these DIF derivatives strongly inhibited the growth of MRSA and VAR, such as *E. faecalis* and *E. faecium*, and suppressed the growth of the other Gram-positive bacteria, such as *S. aureus* and *B. subtilis*, at MICs within the sub-micromolar to the low-micromolar range. In contrast, none of these DIF derivatives had any significant effect on the growth of the Gram-negative bacterium *E. coli* (Kubohara et al. 2019). Ethanol, methanol and dichloromethane extracts of six myxomycete species, isolated from turkey, were tested on bacteria and fungi. Three kinds of extracts from *Symphytocarpus amaurochaetoides* (Fig. 8c), *Lindbladia tubulina* (Fig. 9a), *F. septica and Tubifera ferruginosa* (Fig. 9b) had antibacterial and antifungal effects. But these extracts from *Stemonitis fusca* (Fig. 9c) *and L. epidendrum* had lower antimicrobial activity (Baba et al. 2020).

## The distribution pattern of the different slime molds producing antibiotic compounds

**Fig. 10:**
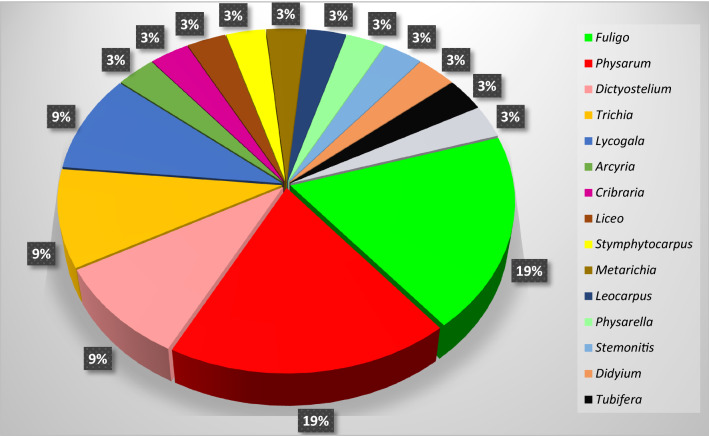
The pattern of distribution of the reported slime molds producing antimicrobial agents

In this review, I summarized the all reports related to slim molds producing antibiotic compounds. These include 31 slime molds with different agents, and some of them were crude extracts. It is noteworthy that only two genera of *Myxomycetes*, *Fuligo*, and *Physarum*, involve 38% of total slime molds studied as antimicrobial sources. Three other frequent genera, *Dictyostelium, Trichia, and Lycogala,* each with 9% of frequency, involve 27% of total slime molds producing antibiotics. In addition, these five genera, include 65% reported. The remaining 35% of the slime molds producing antimicrobial agents are scattered across 11 other genera, each one 3%. Figure 10 shows the distribution pattern of these slime molds. Except for one gene, *Dictyostelium* a cellular slime mold, the other genera are Myxomycete, true or acellular slime molds. Because these creatures usually present and sometimes abundant in terrestrial ecosystems and are the only macroscopic slime molds, so they found easier (Dembitsky et al. 2005). So only 9% of isolated antibiotics, are from cellular slime molds and 91% are from acellular slime molds.

## Pigments of slime molds are predominant in antimicrobial activity

**Fig. 11:**
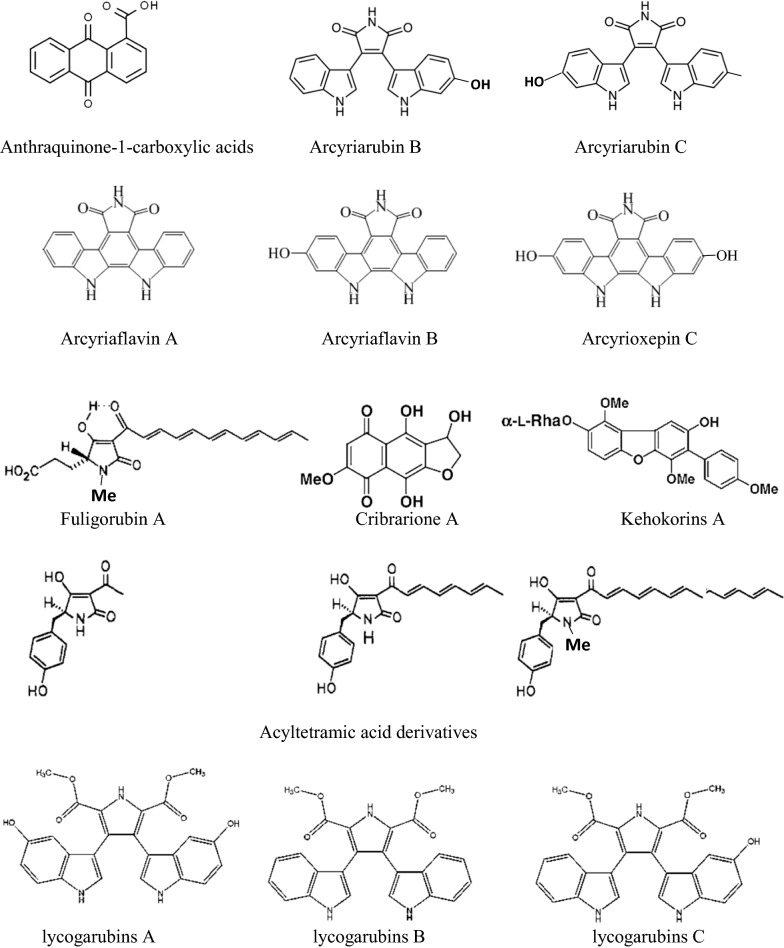
Pigment components from slime molds with antimicrobial activity

According to Table 2, 39.53% of isolated antimicrobial agents from slime molds are crude extracts (involve EPS) and 60.46% are pure agents. 26 pure antimicrobial agents were isolated from slime molds. Figure 11 shows the chemical structure of 17 compounds of these pure antimicrobial agents. It is significant that all of these compounds are pigments, so 65.38% of isolated pure agents are pigments of slime molds and this high dependency ratio is a valuable guide in the antimicrobial agents’ discovery field. Other microbial pigments, such as flavins, melanins, quinones, violacein carotenoids, indigo, and monascins from fungal and bacterial sources, also serve as antimicrobial agents against a wide range of pathogens (Rao et al. 2017). The mechanism of antimicrobial activity of some microbial pigments has been made. For example, Prodigiosin of *Vibrio* sp. DSM 14379 has an effect on *E. coli* with decreasing respiration, inhibition of protein and RNA synthesis, and membrane leakage (Danevcic et al. 2016).

**Table 2:** Antibiotics from slime molds

Antibiotic name	Slime molds	Reference
Anthraquinonic acids (acyltetramic acids)	*Fuligo septica*	Loquin and Prevot (1948)
Mucous secretions or aqueous extracts of plasmodium	*Liceo flexuosa*	Sobels (1950)
Plasmodium extract	*Physarum gyrosum*	Considine and Mallette (1965)
A butanolic and fractionated (pure heterocyclic antibiotic D-1) extract of plasmodium	*Physarum gyrosum*	Schroeder and Mallette (1973)
Arcyriarubins (B, C), arcyriaflavins (B, C), arcyrioxepin A	*Arcyria denudata*	Steglich et al. (1980)
Fuligorubin A (acyltetramic acids)	*Fuligo septica*	Casser et al. (1987)
Vesparione (naphtha[2,3-b]pyran dione derivative)	*Metatrichia vesparium*	Kopanski et al. (1987)
Acyltetramic acids	*Leocarpus fragilis*	Steglich (1989)
Lycogarubins A-C (dimethyl pyrroledicarboxylate)	*Lycogala epidendrum*	Hashimoto et al. (1994)
Methanolic extract of fruiting body	*Fuligo septica*	Chiappeta et al. (1999)
AB0022A	*Dictyostelium purpureum K1001*	Sawada et al. (2000)
Bahiensol (A glycerolipid)	*Didymium bahiense* var*. bahiense*	Misono et al. (2003)
Cribrarione A (A dihydrofuranonaphthoquinone)	*Cribraria purpurea*	Naoe et al. (2003)
Fatty acids with Δ^5,9^-position of two double bonds (two)	*Trichia Favogina* *Trichia varia*	Dembitsky et al. (2005)
Fulicineroside (A glycosidic dibenzofuran)	*Fuligo cinerea*	Rezanka et al. (2005)
Kehokorins A	*Trichia favoginea* var. *persimilis*	Kaniwa et al. (2006)
Plasmodial extract	*Physarella oblonga*	Herrera et al. (2011)
Plasmodial extract	*Physarum melleum*	Herrera et al. (2011)
Crude extract	*Fuligo septica*	Jiang et al. (2014)
4-methyl-5-pentylbenzene-1,3-diol (MPBD)	*Dictyostelium discoideum*	Murata et al. (2016)
Lycogalinosides A and B	*Lycogala epidendrum*	Wang et al. (2017)
EPS EPS	*Physarella oblonga* *Physarum polycephalum*	Huynh et al. (2017)
Ethanol, methanol and dichloromethane extract	*Physarum album (Bull.)*	Sevindik et al. (2018)
Differentiation-inducing factors (DIFs)	*Dictyostelium discoideum*	Kubohara et al. (2019)
Ethanol, methanol and dichloromethane extracts	*Symphytocarpus amaurochaetoides* Nann.-Bremek., *Lindbladia tubulina Fr.,* *Fuligo septica* (L.) FH Wigg., *Stemonitis fusca* Roth., *Tubifera ferruginosa* (Batsch) JF Gmel., *Lycogala epidendrum* L. Fr	Baba et al. (2020)

**Fig. 12:**
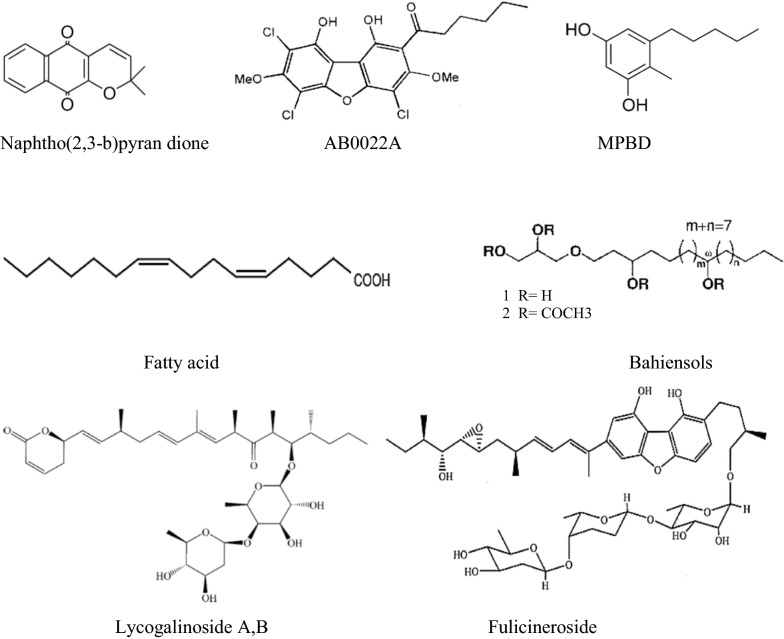
Non-pigment components from slime molds with antimicrobial activity

In Fig. 12, also illustrated non-pigment pure antimicrobial agents from slime molds. Two kinds of compounds, Fatty acid and bahiensols derivatives, had no cyclic structure. The other pure antimicrobial agents from slime molds are cyclic structures. Natural aromatic and cyclic chemicals are in great demand in different fields such as pharmaceuticals, food, perfumes, and cosmetics. For example, the antimicrobial activity of various essential oils from plants, with aromatic structure, reported many times (Androutsopoulou et al. 2021). This point also could be a guide in drug discovery fields.

## Challenges and perspectives

The first antibiotic, penicillin, was identified in the fungus *Penicillium notatum.* Since that discovery, most of the antibiotics identified, have been isolated from fungi and Actinobacteria (Kubohara et al. 2019; Durand et al. 2019). However, the emergence of drug-resistant bacteria, such as MRSAs and VREs, has been attributed to the intensive use or the use of subtherapeutic dosing of antibiotics in medicine. Therefore, searches for bio-resources that produce novel antimicrobials or new antibiotic molecules from old sources are required. However, the investigative studies of slime molds expressed above indicated that these protists have a special life cycle and are estimated to produce many valuable secondary metabolites with unique chemical structures. It has been predicted that these metabolites could represent a potential source of natural active products, such as antibiotics, which offers a broad field of further studies (Kubohara et al. 2019; Kubohara and Kikuchi 2019; Sasaki et al. 2020)^.^

Table 2 provides the process of these investigations. As mentioned, only a few known species of slime molds have been investigated so far. This is due to the difficult cultivation of slime molds. Therefore, Sihui and collaborates mentioned that for further progress in isolation of the bioactive compounds, the development of cultivation strategies to grow slime molds is crucial. They provided different culture methods including pure culture, feeding culture, liquid culture, moist chamber culture and hanging drop culture. But all methods have disadvantages. In pure culture, most slime molds do not grow on laboratory medium. In feeding culture, which compared to pure culture, bacteria or yeast are added to the medium as food, the plasmodia are often mixed with bacteria and yeasts. Liquid culture is not suitable for most slime molds. In the moist chamber culture method, the whole life cycle cannot be observed and artificially selected for extraction. The hanging drop method is only for spore germination and is not suitable for the whole life cycle (Li et al. 2020).

Slime molds have a special life cycle and are predicted to produce many valuable metabolites with unique chemical structures. Hence, these protists have been predicted to produce a potential source of natural active products. However, further studies are still needed since the number of species investigated so far is still relatively small. So the biggest challenge for obtaining new antimicrobial compounds from these valuable natural sources is to find effective laboratory culture methods.

## Data Availability

Not applicable.
